# From face-to-face to e-learning: transitioning to new training models to strengthen the health system by supporting primary healthcare workers in low- and middle-income countries

**DOI:** 10.1136/bmjgh-2025-021212

**Published:** 2026-03-16

**Authors:** Christy-Joy Ras, Daniella Georgeu-Pepper, Robyn Curran, Ruth Vania Cornick, Candice Daniels, Cassandra Bassett, André Janse van Rensburg, Elrien Joubert, Makhosazana Lungile Simelane, Lauren Faye Anderson, Pearl Wendy Spiller, Faye Eshraghi, Inge Petersen, Lara R Fairall

**Affiliations:** 1 Knowledge Translation Unit, Department of Medicine, University of Cape Town, Observatory, Western Cape, South Africa; 2 Centre for Rural Health, College of Health Sciences, University of KwaZulu-Natal, Durban, KwaZulu-Natal, South Africa; 3 People Development Centre, Western Cape Department of Health, Cape Town, Western Cape, South Africa; 4 School of Life Course & Population Sciences, King’s College London, London, UK

**Keywords:** health personnel, health systems

## Abstract

Reliance on purely face-to-face in-service training for primary healthcare workers in low- and middle-income countries is increasingly unsustainable. The COVID-19 pandemic accelerated the transition of the University of Cape Town Knowledge Translation Unit’s Practical Approach to Care Kit programme from a facility-based cascade model to online and blended learning formats.

This paper analyses the implementation of this transition across 29 courses between 2020 and 2023 in South Africa. Using the Health System Process Goals framework, we reflect on the challenges and enablers of e-learning, shifting the focus from digital training as a standalone technical solution to a systemic enabler of health system strengthening.

While e-learning expanded access and standardised content, successful implementation relied on addressing systemic barriers. Key learnings include the necessity of subsidised (‘reverse-billed’) data to ensure equitable access; the superiority of a ‘blended’ pedagogical model that combines digital content with peer interaction and in-person technical support and the value of automated reporting for workforce management. The systemic barriers included the lack of protected time for learners, which risks placing an inequitable burden on the workforce and reliance on donor funding, challenging long-term institutionalisation.

For e-learning to effectively strengthen the health system, it must be integrated into administrative workflows and budget lines. We provide actionable recommendations for Ministries of Health, funders and implementers, advocating for a transition to government-owned platforms, accredited blended learning models and policy that mandates protected time for capacity development.

**Summary box**

- E-learning expands access to continuing professional development—essential for keeping the health workforce current and skilled in delivering evidence-informed patient care—but in-person support is required to effectively navigate and implement the transition from traditional face-to-face training to an online format within the health system.
- Advantages of e-learning include the ability to standardise and ensure quality course content, provide access to people in remote settings, track progress of learners, auto-generation of certificates and provision of training reports for management and planning.
- Blended learning has been found to best support course uptake and completion, where there is in-person technical support for registration and start-up, protected time for group learning around online cases as well as consistent support of course progress towards completion.
- Challenges to e-learning include persistent preference for group learning by primary care health workers, negotiation of protected time to complete training, digital literacy, limited internet access, limited evaluation and dependence on external partners and funders.

## Introduction

 Primary healthcare (PHC) is recognised as the most inclusive, equitable, cost-effective and efficient approach to achieve Universal Health Coverage (UHC),^1^ yet it faces the largest shortfall of skilled healthcare workers (HCWs).^2^ Effective, scalable continuous professional development is required to equip primary HCWs with current guidelines and protocols for evidence-informed care.^3^ In PHC settings, challenges such as reaching HCWs based in facilities dispersed across vast areas, staff shortages and high turnover hinder achieving Sustainable Development Goal 3 and UHC.^4^ Addressing these challenges through robust in-service training is imperative to equip HCWs for effective, responsive and sustainable health services.^5^

Over the past 20 years, the University of Cape Town’s Knowledge Translation Unit (KTU) has collaborated with local government in South Africa on a multifaceted PHC health systems strengthening initiative called the Practical Approach to Care Kit (PACK). Since 2013, this programme, also known as Adult Primary Care (APC), has been incorporated into the Ideal Clinic Realisation and Maintenance programme rolled out to all of the country’s 3500 primary care clinics.^6^ The PACK programme has been localised in other countries, including Botswana,^7^ Nigeria,^8^ Ethiopia,^9 10^ Indonesia and Brazil^11 12^ to support PHC reforms. Central to PACK is a 180-page comprehensive, evidence-informed clinical decision support tool, the PACK ‘guide’, for use during primary care consultations and covering an approach to the management of common symptoms and long-term conditions presenting in primary care, integrating guidance on communicable diseases, non-communicable diseases, mental illness and women’ health.^13 14^

A training and implementation strategy supports integration of the guide into everyday practice.^15^ Described in full elsewhere,^15^ training follows a cascade model that capacitates government-employed master trainers to train facility trainers to deliver onsite, in-service training to HCW teams in PHC facilities.^15^ This methodology uses educational outreach^5 16 17^ delivered through multiple 1–1.5 hour face-to-face interactive sessions conducted once or twice weekly at clinics. Embedded adult educational principles promote experiential learning in groups, using clinical case scenarios that closely replicate patient journeys and represent priority health conditions in PHC. On-site delivery allows immediate application in clinical practice between sessions. This on-site training is not a standalone event; it forms part of a broader, ongoing health system strengthening intervention aimed at delivering integrated, up-to-date PHC by as many HCWs as possible, both within clinics, where all HCWs in a facility are trained, and across facilities and health districts.

The PACK implementation model, used for rapid scale-up in South Africa^15^ and Ethiopia,^10^ relies on consistent, high-quality facilitation and sufficient master and facility trainers to maintain the cascade, who in turn require consistent training and supervision. Although studies on the scaled-up intervention show some improvement in quality of care,^18^,^22^ sustaining this model in the resource constraints of a health system beyond the initial implementation period can prove challenging. Training of master and facility trainers requires time away from their regular duties, and even though HCWs are trained on-site at facilities, the delivery logistics can be demanding. High attrition of HCWs, absenteeism, frequent rotation, shift work and large distances between PHC facilities are some of the barriers to successful scale-up and maintenance of this training model.

This paper describes and reflects on the transition from face-to-face in-service training to e-learning and blended approaches, and its implementation in different PHC contexts within South Africa. It forms part of a collection of papers on PACK, which describe its role as part of health system strengthening reforms in low- and middle-income countries (LMICs).

### Transitioning face-to-face training to e-learning

**Figure 1:**
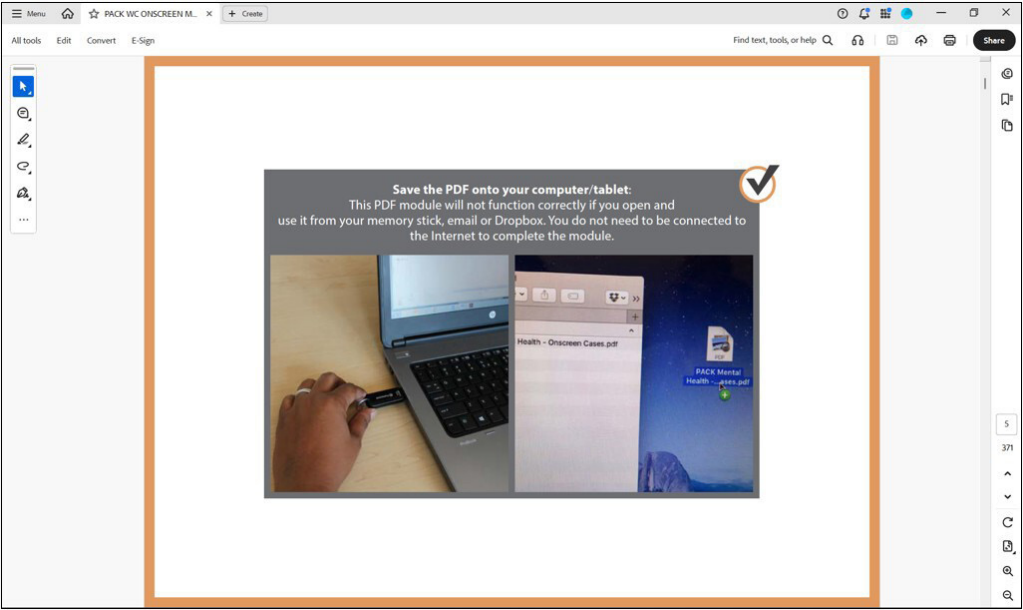
Screenshot of the interactive PDF with instructions on how to use the flash drive and save the document onto the device.

The transition began in 2017 with the scoping of various authoring tools and a learning management system (LMS) (online supplement 1). A digital version of the training was piloted using interactive PDFs distributed on USBs (ie, ‘onscreen’ rather than ‘online’). Challenges included limited digital literacy, computer access and inconsistent facilitator support.^23^ As an example of the extent of digital literacy limitations, instructions on how to insert a USB and access and save a PDF on a desktop computer had to be included in the training (figure 1). Participants suggested a smartphone-friendly, online platform to improve accessibility, which was consistent with other reports from South Africa^24^ and Rwanda.^25^ As mobile connectivity per se was not a major challenge, we did not pursue funding infrastructure for offline access, like Raspberry Pi^26^ devices. An LMS facilitating interaction with the PACK guide (embedded as images within quizzes) was needed. Limited options set up to support this type of learning were available at the time. Two options that we had identified were Thinkific^27^ and Moodle.^28^

During the first COVID-19 lockdown, a rapid shift to e-learning was needed. Thinkific was selected for its cost-effectiveness (US$948/year in 2020), user-friendliness, mobile compatibility and offered interactive features suited to our training needs. An example of the traditional paper-based case facilitation format and the e-learning interactive format demonstrates the integrity of the approach in how the same case is experienced (online supplemental file 2). Table 1 compares the key considerations of implementing the different PACK training models, including logistics/distribution, access, technical requirements, user experience and reporting.

**Table 1:** Key considerations for implementation of the different PACK training models

Key considerations	Type of PACK training model		
	Paper-based training	Interactive PDF	Online platform
Logistics/Distribution	Space and time are needed for the onsite session.	Distribution on USB flash drives—needs a laptop or computer.	Links need to be sent via email or messenger apps on mobile phones.
Access	Onsite sessions need to be set up and run by the facility trainer, with staff available to attend.	Offline access.	Internet access necessary—Wi-Fi or data.
Technical requirements	N/A	Basic technical literacy required.	Fair technical literacy required.
User experience	Dependent on skill/confidence of the facility trainer to create engagement.	On screen, limited interactivity, but intended to be a group discussion.	Engaging interactivity, option for both self-directed learning and group work.
Reporting	Attendance registers must be signed, recorded and reported, after which certificates can be generated and issued.	Attendance registers need to be signed, recorded, reported then certificates generated and issued.	System tracks progress and automatically issues certificates. Real-time organisational reports directly from the database.

N/A, not applicable; PACK, Practical Approach to Care Kit.

E-learning was introduced across various projects in different local settings during the period 2020–2023. Initial courses focused on screening, diagnosis and management of COVID-19 in primary care and were used by HCWs in the Western Cape, South Africa purely in an e-learning format, as this corresponded to the first lockdown periods of the pandemic. In late 2020, we piloted hybrid implementation (online and in-person) of a course integrating the screening for and diagnosis of COVID-19 and tuberculosis in 10 primary care clinics in a rural district of the KwaZulu-Natal province and evaluated it in a research project.^22^ Throughout 2021, we used the platform to equip clinicians, pharmacists and researchers working on a large implementation study of the Ad26.COV2.S vaccine, which provided first doses to half a million health workers before South Africa’s third COVID-19 wave,^29^ and 250 000 boosters before the fourth wave.^30^ During 2021, the Eastern Cape became the first province in South Africa to move to a hybrid model for PACK/APC training with widespread uptake even in remote rural areas.

Due to high mobile data costs, during the 2020–2023 period, a service provider was paid to reverse the charge of data cost to the KTU, allowing HCWs free access to courses. This system was possible for major cellular network providers but excluded smaller network providers. In some instances, individuals would convene in venues where free Wi-Fi was available to complete training.

By February 2023, we published 29 online courses covering COVID-19 management in PHC (n=12), PACK/APC (n=11) and six courses to support COVID-19 research^2229^,^31^ (table 2). The courses included multiple update courses on changing management of COVID-19; enrollees were automatically notified of changes via email. Most courses targeted nurses who staff South Africa’s PHC clinics, although some courses were developed for doctors and others for health science students. Some courses were prerequisites for further training, or completion was required to meet criteria to operate a research vaccination site. In South Africa, nurses working in public sector primary care clinics are required to complete PACK/APC training and, at any given time, at least 80% of clinical nursing staff must show evidence of training completion.^6^ This is assessed during quarterly clinic assessments, and certificates of training completion are central to documentation. Thus, automated certificate generation was highly valued.

**Table 2:** Course enrolments and completion rates: March 2020 to February 2023

Course type	Number of courses	Enrolments	Completion rate (%)
Research related	6	4840	73
PACK/APC	11	16 864	55
COVID-19	12	12 727	74
All courses	**29**	**34 431**	**63**

APC, Adult Primary Care; PACK, Practical Approach to Care Kit.

To evaluate the training, the Kirkpatrick model was used, which has four levels of evaluation: level 1—reaction; level 2—learning; level 3—behaviour; level 4—results.^32^ Feedback questionnaires were provided and asked about general perceptions of the course (level 1), any technical difficulties or errors identified and any clinical questions that arose. Quiz scores tracked knowledge acquisition (level 2). Course surveys captured participant feedback on relevance to their practice and behaviour change (level 3). Results (level 4) were not explicitly captured but are discussed in the ‘Discussion of challenges and learnings’ section of the paper.

Course feedback is aggregated monthly to monitor quality and learner satisfaction. For new courses, developers review feedback after 3 weeks to allow for prompt identification and resolution of critical issues, while also capturing initial suggestions for future improvements. Email responses are sent to all those who identified technical difficulties, errors or had additional clinical queries. All courses were published in English.

The mode of capturing and format of reporting training changed from manual to automatic over this transition. Thinkific reports were integrated via an application programming interface (API) with a custom database of facilities, delivering facility-level reports for managers and trainers to identify staff requiring support.

### E-learning uptake

Between March 2020 and February 2023, 34 431 enrolments were recorded with a 63% overall completion rate. There were 12 727 enrolments in COVID-19 courses (74% of which were completed); 16 864 in PACK/APC training courses (55% of which were completed) and 4840 research-related course enrolments (73% of which were completed). Low completion rates for APC training reflect the challenge of its 12-hour duration compared with 2–6 hours for other courses (table 2). Courses were completed in personal time or during protected time at clinics. This flexibility made allowances for users' work schedules and other pressures and was critical in facilitating the scale-up of the vaccine course to 600 people across 400 sites throughout South Africa within 5 days.

**Figure 2:**
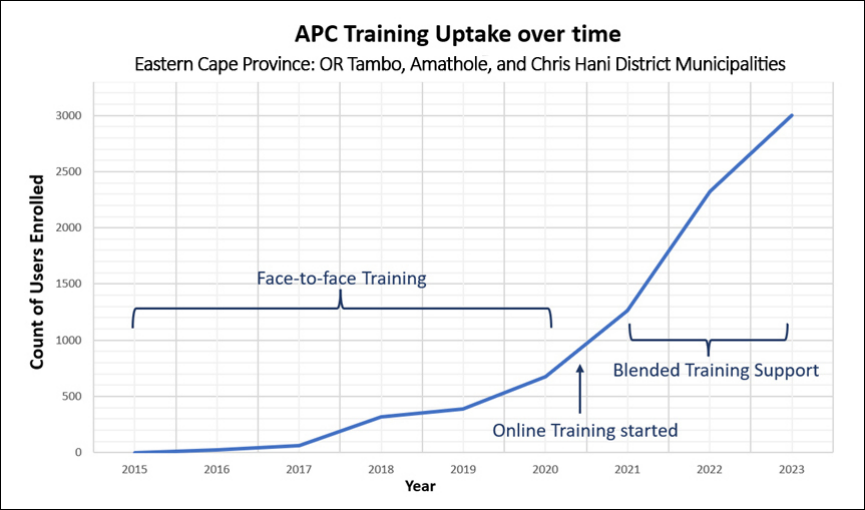
Adult Primary Care (APC) training uptake in 3 municipalities from 2015-2023, showing the impact of the different training modalities.

In the Eastern Cape province, where APC was being actively implemented, e-learning provided a much-needed breakthrough for reaching nurses in remote clinics. Many of these facilities have only one or two nurses and so cannot afford to send them to centralised training courses without disrupting clinical services. Poor road infrastructure and long distances between clinics limited opportunities to provide in-person training. Figure 2 shows the timeline during which we worked with the Provincial Regional Training Centre and a Presidents Emergency Plan for AIDS Relief (PEPFAR) -funded non-governmental organisation to deliver PACK/APC training to nurses over a large expanse and shows the very slow progress over the period 2015–2020, and the large uptake in training that followed introduction and ongoing support of the online APC training courses.

### Discussion of challenges and learnings

Reflecting on the transition from face-to-face to e-learning, we applied the Health System Process Goals framework by Bertone *et al*^33^ to discuss the challenges and learnings in a useful way for future application in the context of e-learning and health systems strengthening. The themes highlight the interplay between digital opportunities and systemic barriers, discussing results of the transition (Kirkpatrick level 4) at a system level.

### Access and infrastructure

A significant barrier to e-learning in LMICs is poor internet infrastructure, unreliable electricity and high data costs.^34 35^ In South Africa, where mobile data are expensive and Wi-Fi in public facilities is scarce, many HCWs rely on personal mobile networks. To mitigate this, we subsidised a service that reversed the data cost to the KTU, enabling participants to access courses without incurring individual expenses. This service, however, was limited to major network providers and was available only while funding permitted. This intervention directly addressed the Learning and Resilience health system process goal (HSPG) of ensuring that the ‘system is able to respond to changes in context’,^33^ specifically by enhancing the capacity to absorb shocks, such as the COVID-19 pandemic, and to maintain essential services, including training, despite infrastructural deficits.

Closely linked to infrastructure is the challenge of digital literacy. Limited digital skills are a well-documented barrier.^36^,^38^ We found that in-person technical support—assisting with email setup, password management and browser settings—was essential, particularly for those new to online learning.^22^ Despite these hurdles, the transition to^39^ online platforms offered a flexibility that face-to-face training could not. Previously, staff shortages and rotation often prevented HCWs from attending fixed in-person sessions. The e-learning format enabled early adopters and those who missed sessions to complete training at their own pace—a feature particularly valued during the COVID-19 lockdowns. This aligns with ‘strengthening capacity at individual, organisational and system levels’, another Learning and Resilience HSPG.^33^

### Pedagogical adaptation

E-learning formats enabled standardisation of training, addressing concerns regarding trainer competence and fidelity to the programme.^40 41^ Affirming the ‘service delivery’ HSPG of ‘quality of services is ensured, providing safe, appropriate, respectful and person-centred care’, the use of interactive quizzes and intentional instructional design in the courses ensured that all learners received engaging, high-quality content, regardless of their location. It is worth noting that this shift altered the role of the facility trainer from a primary facilitator to a co-ordinator focused on supporting enrolment and completion of training.

We learnt that while self-directed e-learning ensures standardisation, it risks isolating learners. Our experience affirms that HCWs often prefer group discussion and case-based learning over solitary computer-based methods.^41 42^ In the KwaZulu-Natal study^22^ and in the Eastern Cape, a ‘blended learning’ approach emerged organically: HCWs completed e-learning modules together, facilitating real-time discussion and local application. This blended model—combining standardised e-learning content with peer engagement—appears to offer the most effective learning experience.^42^,^46^ Implementing this approach aligns with the ‘Learning and Resilience’ HSPG of ‘teamwork and collaboration are supported’ and ‘strengthening capacity at individual, organisational and system levels’.^33^

Furthermore, as e-learning becomes normative, rigorous evaluation mechanisms are critical. While our small quasi-experimental study provided reassurance of effectiveness,^22^ routine evaluation must expand beyond current user feedback of impacts on individual learning and application in practice to wider health systems impacts as Kirkpatrick’s model recommends.^32^

### Institutionalisation

Sustainability requires e-learning to be integrated into the health system’s administrative workflows. Automating the arduous manual process of issuing certificates was a major enabler. Immediate certificates were highly valued by HCWs for facility assessments and continuing professional development recognition. While most LMS platforms allow tracking individual progress, there is minimal reporting functionality at the level of health facilities.^47 48^ Yet, monitoring numbers of trained HCWs is critical for managers who are responsible for managing teams with high turnover. To meet this reporting requirement, Thinkific reports were integrated with a custom reporting database of facilities and staff via API. Detailed, facility-level location-specific reports for managers and trainers to identify who needs support to complete training are available, thus enabling targeted capacity-building. This feature of e-learning in the health system aligns with the ‘Use of Resources’ HSPG, which states that ‘Monitoring systems are integrated, adapted to local needs and user friendly and routine health information systems are supported to be timely, complete and accurate’.

However, long-term institutionalisation faces the challenge of sustainability—highlighting the need for the first HSPG of ‘ownership by stakeholders at different levels of the health system and coordination of external stakeholders/donors are emphasised’.^33^ This has been done by the South African National and Western Cape Provincial government, but LMS platforms in LMICs are often donor-funded rather than supported through domestic budget lines. To fully realise the ‘Use of Resources’ goal where ‘funding is based predominantly on public or compulsory sources’, platform ownership must transition to government agencies, ensuring sustainability beyond project cycles.

### Equity and policy support

The transition to e-learning raised equity concerns regarding the use of time; participants reported a lack of protected time for e-learning. The expectation of after-hours e-learning completion contradicts the HSPG ensuring ‘Human resources are deployed where needed, with the right skills, attitude and support’,^33^ which includes motivating working environments and retention efforts. In South Africa, expecting a predominantly female nursing workforce with significant domestic responsibilities to train during personal time is neither feasible nor equitable.^49^ While the flexibility of the online platform allowed learners to accommodate work pressures, it is essential that this flexibility does not translate into an uncompensated burden on the workforce. To align with the goal where ‘Stakeholder participation [is] promoted’, the voices of the workforce must be heard, and management must support protected time with policy. Without this, the system fails to foster a ‘Culture of service, commitment and solidarity’, as the burden of system strengthening is placed unfairly on the individual’s personal time.

Table 3 lists these lessons and highlights the implications for stakeholders adopting the use of e-learning in an LMIC context.

**Table 3:** Lessons learnt and stakeholder implications for adopting the use of e-learning in an LMIC context

Lessons learnt	Implications for implementers	Implications for policymakers	Implications for funders
**Pedagogical adaptation**: While online content ensured standardisation, HCWs preferred group interaction. A ‘blended’ model emerged naturally where teams consumed digital content together to discuss local application.	**Adopt a blended approach:** use digital platforms to deliver core knowledge but organise in-person or virtual focus groups for clinical application and peer support.	**Accredit blended learning:** update CPD frameworks to recognise hybrid models (group work and digital) as valid learning hours.	**Personnel and facilitation support:** do not restrict funding to software development; allocate resources for the training of facilitators and mentors required to support the blended approach of learning.
**Access and infrastructure**: High mobile data costs and low digital literacy (eg, struggles with passwords/browsers) were major barriers requiring hands-on technical support and services to ‘reverse-charge’ the data to the KTU instead of the individual.	**Design for low-resource environments:** consider ‘offline-first’ mobile applications, depending on connectivity, and provide onsite ‘digital onboarding’ support to help users navigate basic technical hurdles.	**Negotiate zero-rating:** leverage government influence to negotiate ‘reverse-billed’ or zero-rated data access for educational health platforms with telecommunication providers.	**Budget for access:** explicitly include line items for mobile data costs and IT support personnel in grant budgets, acknowledging that content is useless without access.
**Equity and policy supports**: Expecting the nursing workforce to complete training after hours was found to be assumptive and due to significant domestic responsibilities, proved inequitable.	**Flexible scheduling:** promote training models that allow for ‘bite-sized’ learning that can be completed during short, regular sessions rather than requiring long blocks of time.	**Mandate protected time:** issue circulars or policy directives that classify e-learning as official work duties, requiring facility managers to roster protected time for study during working hours.	**Align metrics with reality:** avoid setting grant targets that are not aligned with capacity; support programmes that integrate training into service delivery models.
**Institutionalisation**: Automated digital tracking and certificate generation solved administrative backlogs, but sustainability is at risk as platforms are often donor-funded rather than government-owned.	**Ensure interoperability:** build systems with APIs that can feed training data directly into national HRH databases for real-time workforce monitoring.	**Budget for sustainability:** transition digital training platforms from ‘special projects’ to line items in the Ministry of Health’s annual Information, Communication and Technology and HRH budgets.	**Plan for exit:** require a ‘transition to government’ plan at the start of funding cycles, ensuring platforms are compatible with government servers and regulatory standards.

CPD, continuing professional development; HCW, healthcare worker; HRH, Human Resources for Health; KTU, Knowledge Translation Unit.

### Ongoing development and evaluation of e-learning

The KTU’s e-learning offerings have continued to expand, serving as a critical health system catalyst for evidence-based, localised and policy-aligned clinical training. Operating three online schools serving South Africa and Brazil,^11 12^ with over 100 courses, the KTU supports integrated service delivery. Courses cater to all cadres—from community health workers to security guards at facilities to doctors—strengthening human resources beyond just clinical staff. We have successfully incorporated artificial intelligence to streamline course development and manage feedback and continue to learn and grow in this space. The recorded 71 750 enrolments and 47 881 completions by end-2025 represent a 67% completion rate, significantly higher than the reported average of 10%,^50^ directly impacting systemic capacity and workforce readiness. Research is underway to understand the translation of adult learning principles, many of which depend on in-person and group learning opportunities, to online platforms and their impact on knowledge and clinical practice among PHC HCWs.

## Conclusion

While the appetite for e-learning among PHC HCWs in LMICs is evident, our experience confirms that successful implementation requires shifting from viewing digital training as a standalone solution to integrating it as a systemic enabler of Health System Process Goals for health system strengthening. As summarised in table 3, achieving quality service delivery necessitates a pedagogical shift to a ‘blended’ model, supporting in-person facilitation alongside software to reinforce digital content with peer interaction. Furthermore, equitable resource deployment requires mandating protected time and data budgets, preventing administrative and financial burdens of training from falling on the individual. Ultimately, for these interventions to be resilient and sustainable, they must transition from donor-funded projects to government-owned assets—where interoperable systems and recurring budget lines support long-term institutionalisation rather than temporary, project-based relief.

## Supplementary material

10.1136/bmjgh-2025-021212 online supplemental file 1

10.1136/bmjgh-2025-021212 online supplemental file 2

## Data Availability

No data are available.
