# Supplementary material for: From face-to-face to e-learning: transitioning to new training models to strengthen the health system by supporting primary healthcare workers in low- and middle-income countries
Source: BMJ Glob Health. 2026 Mar 16;9(Suppl 3):e021212. doi: 10.1136/bmjgh-2025-021212 (PMC13266243; doi:10.1136/bmjgh-2025-021212)
Supplement: online supplemental file 1 [file bmjgh-9-Suppl_3-s001.pdf]

Supplementary File 1: Scope of eLearning Software and Tools

13 October 2017

	Articulate 360	PDF cases	Adobe Captivate	Adobe Animate
**Products**	Articulate is a suite of applications or tools. The most popular applications are:<br><br>• Storyline 360: Interactive courses Examples of courses created in Storyline: https://community.articulate.com/articles/best-of-2016-the-top-10-most-inspiring-storyline-2-examples<br>• Rise: To develop "responsive courses" Examples of courses created in Rise: https://community.articulate.com/articles/6-inspiring-rise-examples-that-raise-the-bar-for-mobile-e-learning<br>• Products used when starting from PowerPoint, or putting together quick presentations. This means that PowerPoints can be imported into the applications for easy use regarding creating course material.<br>• Articulate Review: An easy way to collect feedback from stakeholders. What the video for further explanation: https://articulate.com/360/review	An interactive PDF.	Adobe Captivate is a program/application that is a part of the Adobe Suite.<br><br>Example of an eLearning course in adobe captivate: http://library.elearningbrothers.com/product/9352748-Negotiation-Skills	Adobe Animate is a program/application that is a part of the Adobe Suite.
**Display on different devices**	Yes (Articulates methods can be glitchy)<br><br>Build Interactive E-Learning with Storyline 3<br><br>Smartphones would need to:<br>- Be able to access the internet	Basic functionality and display on mobile devices<br><br>Hotspots, forms, and video will not work on iPads and mobile devices. This is due to the different readers that have been standardised on different devices.	Yes	Yes

	- Access to the app store<br>- Have Articulate mobile player app (only for articulate)			
**Comparisons**	• Easy to use.<br>• Large online community.<br>• Unlimited access to a content library.<br>• Less design friendly (a lot like PowerPoint – Can only create templates through a Slide Master). Therefore, it will take longer to produce designs.<br>• Quizzes and tests can combine multiple results for a final score; allowing for users to get a full idea on their performance.<br>• Courses can be translated for learners across the globe. Supports right to left languages and various character sets.<br>• There is the ability to include an interactive glossary.<br>• Unable to work with Adobe Suite and unable to import PDFs into interface. Can Import PowerPoints.	• Easy to use.<br>• No online community.<br>• Create our own content.<br>• Design friendly<br>• Unable to calculate test results.<br>• Would need to source a translator or leave in English.<br>• Technical restraints in terms of navigation (unable to create a glossary).	• Steeper learning curve.<br>• Lack of a community.<br>• Asset store.<br>• Design friendly – Makes use of Master slides and style sheets.<br>• Quizzes and tests can combine multiple results for a final score.<br>• Would need to source a translator or leave in English.<br>• Can add a glossary<br>• Works with Adobe Suite, imports PDFs and PowerPoint.	• Need to refresh knowledge.<br>• Lack of a community.<br>• No asset store.<br>• Design friendly (medium)<br>• Simple scoring.<br>• Would need to source a translator or leave in English.<br>• Can add a glossary<br>• Works with Adobe Suite, imports PDFs.
**Interactivity & functionality**	• Can make use of gamification.<br>• Buttons, dials, sliders, and markers<br>• Touch screen gesture support<br>• Does not have GPS support<br>• Click, hover, and drag functions<br>• Contains a "read-only" seek bar. Therefore, users are unable to skip through information but can keep track of how far they are within the process.<br>e.g.	• Buttons (Next buttons)<br>• Back buttons: Time consuming (Need to link each to a specific page number)<br>• Correct answer buttons (Linked to specific page numbers)<br>• Video (Can't function on mobile devices)<br>• Audio<br>• Forms (need to be online to submit to a URL)	• Can make use of gamification<br>• Buttons<br>• Touch screen gesture support<br>• GPS support - Create assessments that require learners to be at a particular geo-location. Set location parameters easily using the integration of Google Maps. Learning material can be customised to a specific location. Therefore, depending on the location of the user, the text, style, and people in the template will change to match.	• Can make use of gamification<br>• Buttons<br>• Touch screen support<br>• Click, hover, and drag functions (responsive)<br>• Video/animation<br>• Audio

	https://www.youtube.com/watch?v=vpkmKbw4Fm8<br>• Screencasting – Also known as video screen capture. It records what a user is doing on their computer. This comes in handy when explaining to users how to use a software product as the user can see what buttons to click for certain functions and where to find those buttons etc on the interface.<br>• Adobe flash input<br>• Video<br>• Audio		Further explanation: https://elearning.adobe.com/2016/05/new-geolocation-enabled-scenario-and-interaction-templates-added-to-adobe-captivate-assets/<br>• Click, hover, and drag functions (responsive)<br>• Does not have a "read-only" seek bar<br>• Screencasting<br>• Flash input<br>• Video<br>• Audio	
**Web connection**	Online and offline (occasional connectivity)	Online and offline (functions better offline)	Online and offline	Online
**Data collection and Analysis**	• Tin Can API 1.0: Also known as Experience API (xAPI) is a new specification for learning technology that makes it possible to collect data about the wide range of experiences a person has (online and offline). Internet connection is stored in an LRS (Learning Record Store) therefore information can be stored and tracked with occasional internet connection. https://www.youtube.com/watch?v=y42MSS1DJqc<br>It can be used with an LMS system such as Moodle. https://tincanapi.com/overview/<br>• SCORM: Sharable Content Reference Model. SCORM governs how online learning content and Learning Management Systems (LMSs) communicate with each other. It is	Analogue methods of data and collection analysis.<br><br>This means that people report to their FT on their completion and progress in *person*, it isn't done through an LMS.<br><br>Although this method of data analysis is useful. Combining analogue and digital methods of data collection would be of greater value for data analysis. This is because both methods allow for important qualitative and quantitative data aspects.	• Tin Can API / xAPI<br>• SCORM<br>• AICC	• Does not publish Tin Can API (but could make use of Captivate/Articulate to publish to Tin Can API)<br>• SCORM<br>• AICC

	purely a technical standard. SCORM allows educators to track such things as learner completion of a course, pass/fail rates, and how long a learner took to complete a lesson or course. Makes use of internet connection only.<br>• AICC: Aviation Industry CBT Committee. It uses an HTML's HTTP to send information to the LMS and the LMS sends information back to the course as a simple text string. This method is not ideal as it requires full internet access to communicate with the LMS.<br>• JavaScript: A programming language run by most modern web browsers. It supports object-oriented programming, meaning that it is code used to aid in interactivity. It is used as code which aids in the communication of the API to LMS.			
**LMS**	• Publish to Articulate Online: A cost-effective way to deliver and track courses. Makes use of internet connection only.<br>Share content with users in three ways:<br>- Through a link which can be pasted into an email or website.<br>- Post a launch button on a website which links them to the content.<br>- Provide users with access to the Articulate Online user portal.<br>Information can be shared publicly or privately.<br>• Can be put into a Moodle LMS	Have been told it can be uploaded into an LMS.	• In-product LMS preview. Get fine-grained control over all aspects of your course, and reduce dependency on your LMS administrator. Automatically preview courses in Scorm.com, to gauge LMS compatibility and get error logs to identify those portions of the course that need attention.<br>• Publish to Adobe Captivate Prime and other LMSs.<br>• Can be put into a Moodle LMS	Can be put on an LMS.

**Price**	• Individual Price: $ 299 = R 4 066.88 per user, first year<br>• Team Price: $ 399 = R 5 427.04 per user, first year<br><br>• Individual Price: $ 499 = R 6 787 per user, annually<br>• Team Price: $ 649 = R 8 827 per user, annually	-	• Subscription: $30 = R409.04 per month<br>• Full licence price (Retail pricing): $1 335,00 = R 18 202.19, annually<br>• Full licence price (Education pricing): $ 349 = R 4 758.48, annually	Already paid for.
**Output**	• SWF: Shockwave Flash format. Requires a flash player and can only be viewed on desktop.<br>• HTML 5 (can be glitchy)<br>• .exe	Interactive PDF	• SWF<br>• HTML 5 (Works perfectly)<br>• Interactive PDF<br>• Native app	HTML 5
**Things to consider:**<br>How much larger the file size would be using Articulate compared to the PDF – this may be an issue when distributing the product. (If we were to make a carbon copy of the current PDF, the file size would roughly be the same size)				

SCORM vs Tin Can API (xAPI)

	SCORM	Tin Can API/xAPI
**Reliability**	Outdated, therefore it is more prone to errors.	Developed more recently, so it is less susceptible to errors. It is also constantly evolving and updating.
**Tracking**	• Limited to tracking desktop learning<br>• Tracks simple data	• Can be used for desktop and mobile learning<br>• Tracks microlearning: Refers to information that is received in small amounts at a time. It is used to save time.

		• An example of how Tin Can API can be used to track learning experiences with Youtube videos.<br>2013-08-20T15:30:44.135 Jeffrey Horne **terminated** 'Gordon Ramsay: How to Cook the Perfect Steak'<br>2013-08-20T15:30:40.343 Jeffrey Horne **played** 'Gordon Ramsay: How to Cook the Perfect Steak'<br>2013-08-20T15:30:40.005 Jeffrey Horne **paused** 'Gordon Ramsay: How to Cook the Perfect Steak'<br>2013-08-20T15:30:39.799 Jeffrey Horne **skipped** 'Gordon Ramsay: How to Cook the Perfect Steak from 02:28 to 00:00'<br>2013-08-20T15:30:38.685 Jeffrey Horne **paused** 'Gordon Ramsay: How to Cook the Perfect Steak'<br>2013-08-20T15:30:38.497 Jeffrey Horne **skipped** 'Gordon Ramsay: How to Cook the Perfect Steak from 02:30 to 02:28'<br>2013-08-20T15:30:38.250 Jeffrey Horne **paused** 'Gordon Ramsay: How to Cook the Perfect Steak'<br>2013-08-20T15:30:38.038 Jeffrey Horne **skipped** 'Gordon Ramsay: How to Cook the Perfect Steak from 02:07 to 02:30'<br>2013-08-20T15:30:37.295 Jeffrey Horne **paused** 'Gordon Ramsay: How to Cook the Perfect Steak'<br>2013-08-20T15:30:37.103 Jeffrey Horne **watched** 'Gordon Ramsay: How to Cook the Perfect Steak from 01:49 to 02:07'<br>2013-08-20T15:30:34.666 Jeffrey Horne **resumed** 'Gordon Ramsay: How to Cook the Perfect Steak'
**Data**	• Completions<br>• Timings<br>• Pass/Fails<br>• Single Scores	• Completions<br>• Timings<br>Can time how long a person had been on a question.<br>• Pass/Fails<br>• Single scores<br>• Serious games<br>• Simulations<br>• Informal learning<br>• Real-world performance<br>• Offline learning<br>• Interactive learning<br>• Adaptive learning<br>• Blended learning<br>• Long-term learning<br>• Team-based learning
**Communicates with an LMS**	• Yes	• Yes, but doesn't have to, it can just make use of an LRS (Learning Record Store) on its own. An LRS is more powerful alongside an LMS.<br>LRS: A data store system that serves as a repository for learning records collected from connected systems where learning activities are conducted.<br><br>**How an LMS and LRS work together:**

		The LMS is not used for tracking purposes but rather falls away into the background to become more of a content delivery/management platform. It can act as a repository for training material; continue to authenticate users; manage development plans and learning paths; deliver content developed directly on the platform.<br><br>Content is supported by the platform is that it talks to either through an internal LRS (if the current LMS has an LRS; this will send information directly in the same system) or a third party LRS (which will pull and retrieve information when it needs them or information can be assessed on the LRS).<br><br>To retrieve certain data, you need to code in what information you would like to obtain.
