# Supplementary material for: From face-to-face to e-learning: transitioning to new training models to strengthen the health system by supporting primary healthcare workers in low- and middle-income countries
Source: BMJ Glob Health. 2026 Mar 16;9(Suppl 3):e021212. doi: 10.1136/bmjgh-2025-021212 (PMC13266243; doi:10.1136/bmjgh-2025-021212)

Supplementary File 2: Examples of paper-based and e-learning PACK Training

Paper-based, case scenario "Connor" (image below,) presented in the instruction template used by a Facility Trainer with a group learning how to use the PACK Guide (see box with dotted lines).

Note: Column 1 scripts questions for the facilitator to ask the group, column 2 provides clinical details for case management to be given as the participants use the PACK Guide, and column 3 lists what page to use in the PACK Guide.

Case 1: Connor (1 visit)

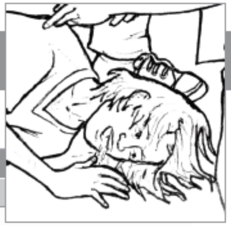

Outline of case:

Symptom	Diagnosis	Routine care	Urgent?	Learning aim	Guide content update
**Introductory cases**					
Fit/seizure	Known epileptic has a prolonged seizure	-	Yes	Introduction, Contents page, red box	**Seizures/fits**<br>• Algorithm revised to align with latest evidence.<br>• New detail added on investigations for a 1st seizure.

Instructions for the facilitator:

- Before you give the participants the case scenario, explain that they will need to use the statements in the guide as prompts to ask you questions about the case.
- Once you have read the case scenario out, use column 1 to ask the participants where they will go in the guide, and to prompt what they should be looking for.
- Only give the required information from column 2 as the participants ask you questions from the guide.
- Follow in the guides with the participants using column 3 to ensure you are on the right page.

Connor, a 24 year old man known with epilepsy, is having a seizure in the waiting room. You are first on the scene.

	1 **FACILITATOR** to ask these questions to prompt using the guide:	2 As the **PARTICIPANTS** use the guide give these details to help answer their questions about the case:	3 Page/s to follow in the **GUIDE**:
**SYMPTOM**			Contents, 8
1	Does the client need urgent attention?		
2	How do you manage this symptom?	• Connor has not been injured.<br>• His glucose is 5.<br>• His relative says he does not drink alcohol<br>• He is still fitting 10 minutes after the first dose of lorazepam.	
3	What page do you go to assess his airway, breathing, circulation and level of consciousness?	• Connor does not respond to your voice<br>• You feel a strong pulse.<br>• He is breathing<br>• His airway is not obstructed and there are no foreign bodies or fluids in his mouth.<br>• He does not have difficulty breathing and is breathing at a normal rate. His lips are pink.<br>• You already have IV access, his BP is 136/88 and pulse 94.<br>• He has no injuries<br>• You assess his GCS: best motor response 5; best verbal response 3; eye opening 1.	5
4	What page do you return to once completing assessment of A,B,C and GCS?		8
5	How do you continue management?	• He is still fitting 5 minutes after the second dose of lorazepam.<br>• His estimated weight is 70kg.<br>• He stops fitting after 25 minutes while on the phenytoin infusion.	
6	Do you need to refer the client?		

TIP: don't give away all this information at once. Follow in the guide and respond to the questions of the participants.

Summarise the case:

Highlight the pathway of the pages/points you used through the guide in order to manage the client.

PACK e-Learning course format

An example of the same case, "Connor", in a quiz format on Thinkific™ that gives the scenario, then poses a question (equivalent to Column 1 on the Case template) with answer options. The following screen (pop out image) is the answers and where to find them (similar to Column 3 in the case template), highlighted on the image of the page in PACK.

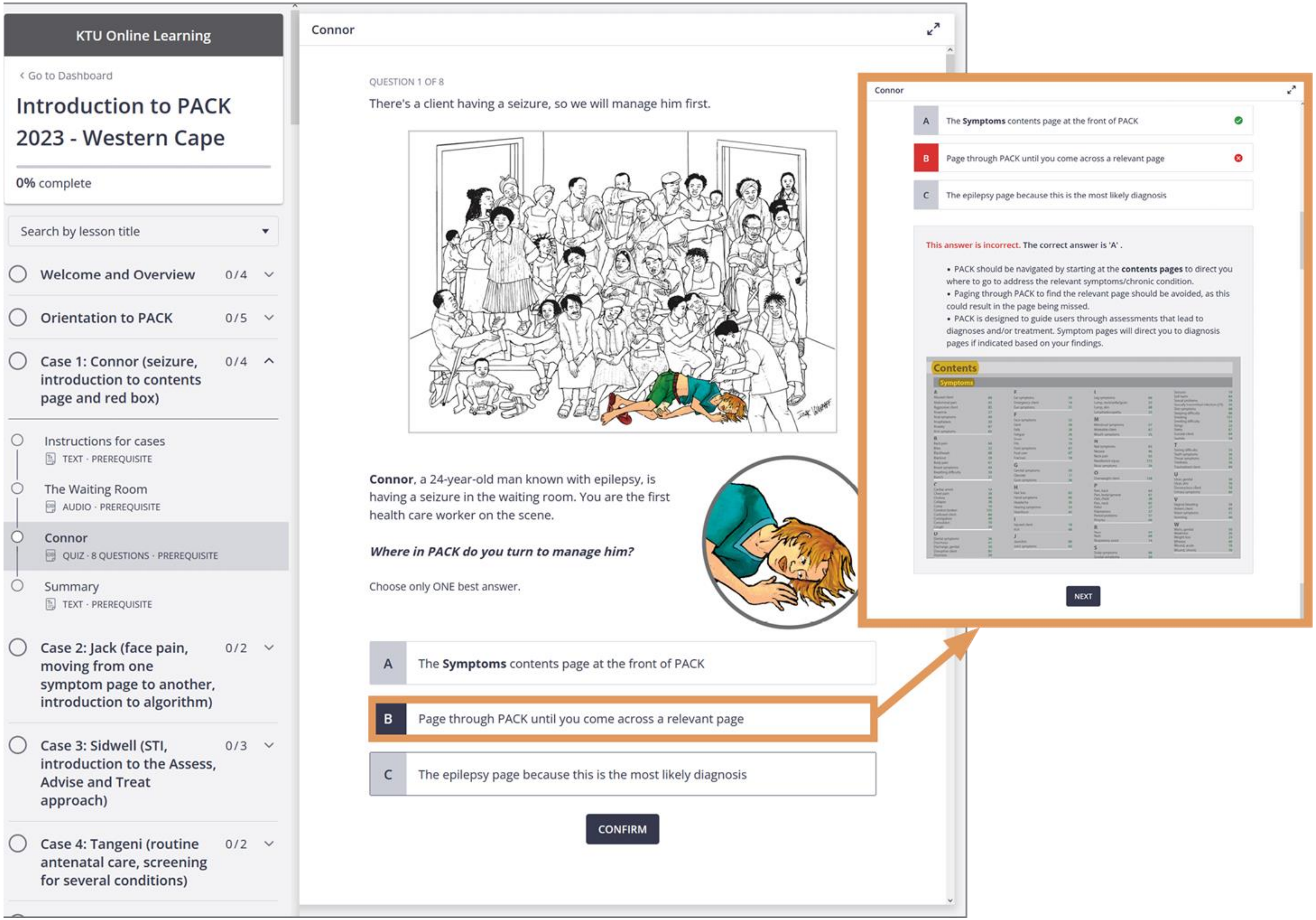

PACK e-Learning course format continued…

In question 5 information is shared to direct the management of the case (equivalent to Column 2 on the Case template), with a question posed on what decision to make next.

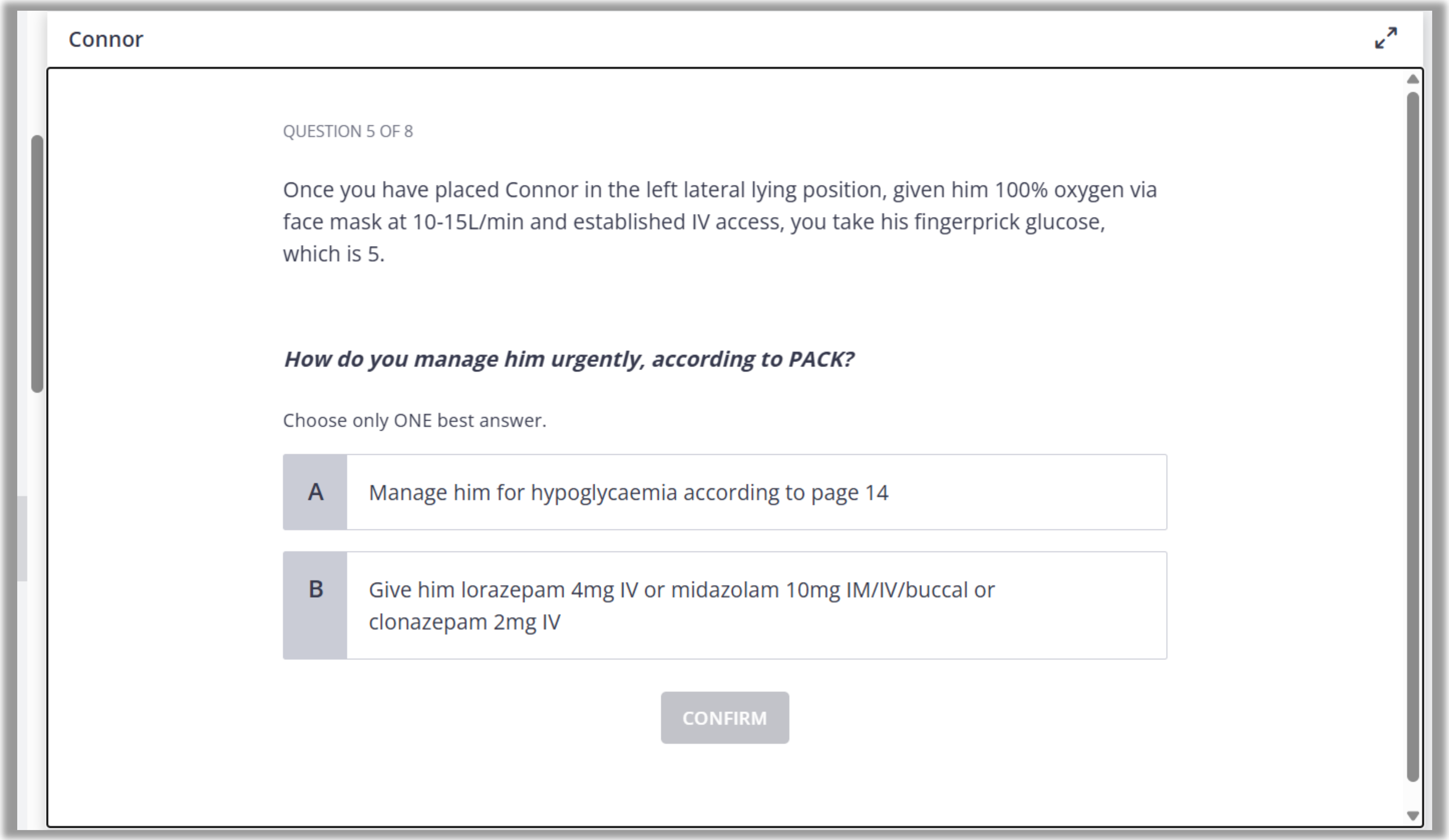

PACK e-Learning course format continued…

Once the answer has been selected immediate feedback is given, with the text reference highlighted in the image of the page in PACK .

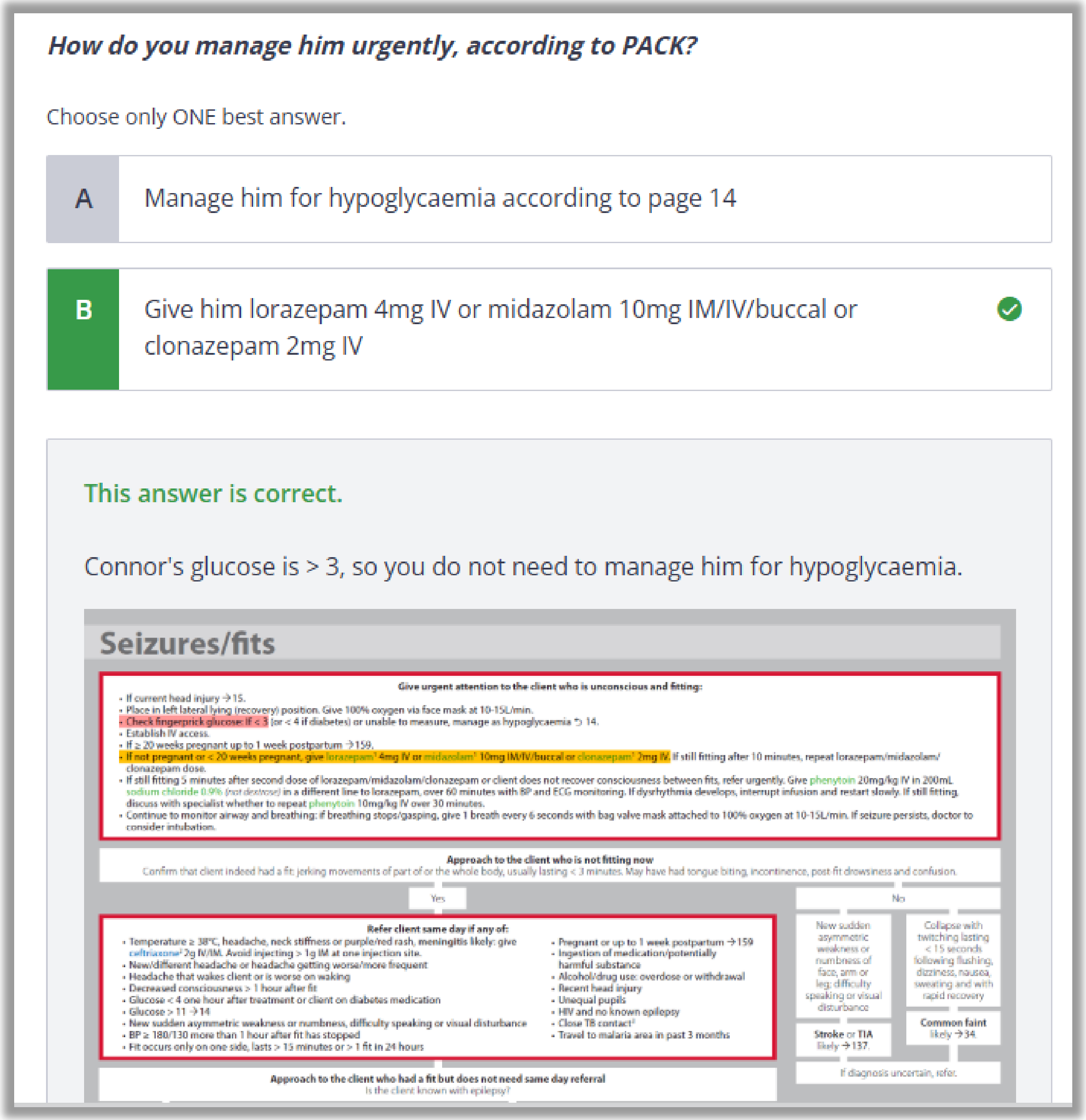

Supplement: online supplemental file 2 [file bmjgh-9-Suppl_3-s002.pdf]
